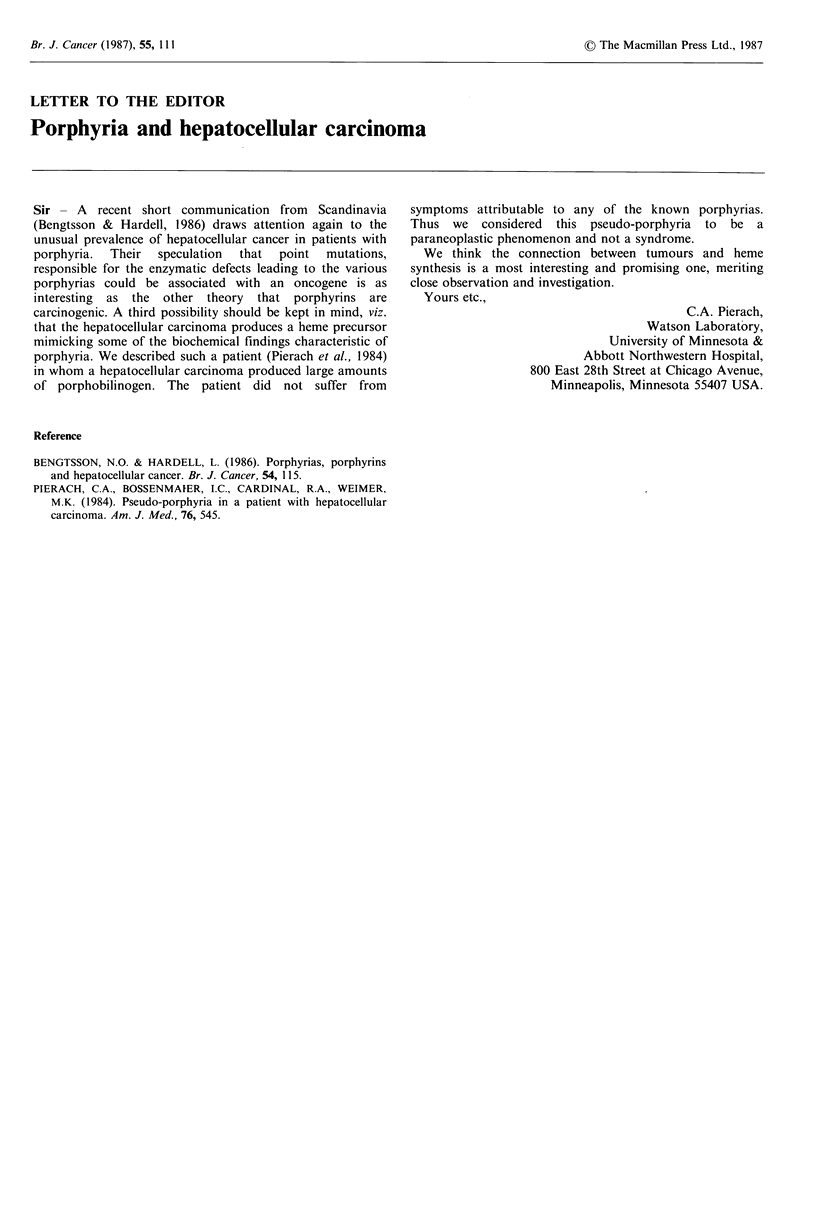# Porphyria and hepatocellular carcinoma

**Published:** 1987-01

**Authors:** C.A. Pierach


*Br. J. Cancer* (1987), **55**, 111 © The Macmillan Press Ltd., 1987

### LETTER TO THE EDITOR

### Porphyria and hepatocellular carcinoma

**Sir** – A recent short communication from Scandinavia (Bengtsson & Hardell, 1986) draws attention again to the unusual prevalence of hepatocellular cancer in patients with porphyria. Their speculation that point mutations, responsible for the enzymatic defects leading to the various porphyrias could be associated with an oncogene is as interesting as the other theory that porphyrins are carcinogenic. A third possibility should be kept in mind, *viz.* that the hepatocellular carcinoma produces a heme precursor mimicking some of the biochemical findings characteristic of porphyria. We described such a patient (Pierach *et al.*, 1984) in whom a hepatocellular carcinoma produced large amounts of porphobilinogen. The patient did not suffer from symptoms attributable to any of the known porphyrias. Thus we considered this pseudo-porphyria to be a paraneoplastic phenomenon and not a syndrome.

We think the connection between tumours and heme synthesis is a most interesting and promising one, meriting close observation and investigation.

Yours etc.,

C.A. Pierach,
Watson Laboratory,
University of Minnesota &
Abbott Northwestern Hospital,
800 East 28th Street at Chicago Avenue,
Minneapolis, Minnesota 55407 USA.

**Reference**

BENGTSSON, N.O. & HARDELL, L. (1986). Porphyrias, porphyrins and hepatocellular cancer. *Br. J. Cancer*, **54**, 115.

PIERACH, C.A., BOSSENMAIER, I.C., CARDINAL, R.A., WEIMER, M.K. (1984). Pseudo-porphyria in a patient with hepatocellular carcinoma. *Am. J. Med.*, **76**, 545.